# PacBio long read-assembled draft genome of *Pythium insidiosum* strain Pi-S isolated from a Thai patient with pythiosis

**DOI:** 10.1186/s13104-023-06532-7

**Published:** 2023-10-13

**Authors:** Theerapong Krajaejun, Preecha Patumcharoenpol, Thidarat Rujirawat, Weerayuth Kittichotirat, Sithichoke Tangphatsornruang, Tassanee Lohnoo, Wanta Yingyong

**Affiliations:** 1 Department of Pathology, Faculty of Medicine, Ramathibodi Hospital, Mahidol University, Bangkok, Thailand; 2 Interdisciplinary Graduate Program in Bioscience, Faculty of Science, Kasetsart University, Bangkok, Thailand; 3 Research Center, Faculty of Medicine, Ramathibodi Hospital, Mahidol University, Bangkok, Thailand; 4 Systems Biology and Bioinformatics Research Group, Pilot Plant Development and Training Institute, King Mongkut’s University of Technology Thonburi, Bangkhuntien, Bangkok, Thailand; 5 National Omics Center, National Science and Technology Development Agency, Pathum Thani, 12120 Thailand

**Keywords:** *Pythium insidiosum*, Pythiosis, Draft genome, Next-generation sequencing

## Abstract

**Objectives:**

*Pythium insidiosum* is the causative agent of pythiosis, a difficult-to-treat condition, in humans and animals worldwide. Biological information about this filamentous microorganism is sparse. Genomes of several *P. insidiosum* strains were sequenced using the Illumina short-read NGS platform, producing incomplete genome sequence data. PacBio long-read platform was employed to obtain a better-quality genome of *Pythium insidiosum*. The obtained genome data could promote basic research on the pathogen’s biology and pathogenicity.

**Data description:**

gDNA sample was extracted from the *P. insidiosum* strain Pi-S for whole-genome sequencing by PacBio long-read NGS platform. Raw reads were assembled using CANU (v2.1), polished using ARROW (SMRT link version 5.0.1), aligned with the original raw PacBio reads using pbmm2 (v1.2.1), consensus sequence checked using ARROW, and gene predicted using Funannotate pipeline (v1.7.4). The genome completion was assessed using BUSCO (v4.0.2). As a result, 840 contigs (maximum length: 1.3 Mb; *N*_50_: 229.9 Kb; *L*_50_: 70) were obtained. Sequence assembly showed a genome size of 66.7 Mb (178x coverage; 57.2% G-C content) that contained 20,375 ORFs. A BUSCO-based assessment revealed 85.5% genome completion. All assembled contig sequences have been deposited in the NCBI database under the accession numbers BBXB02000001 - BBXB02000840.

## Objective

Short- and long-read genome sequencing technologies are now widely available for generating genome data of various organisms [1]. The major difference between the two is the maximum length of generated sequence reads: 150–400 bases for the short-read platforms (i.e., Illumina and Ion Torrent) [2, 3] and many kilobases for the long-read platforms (i.e., Pacific BioSciences (PacBio) [4] and Oxford Nanopore [5]). Another difference is the genome sequence coverage, in which the short-read platforms tend to produce a relatively-higher coverage (although likely incomplete) genome of an organism of interest. In contrast, the long-read platforms generate a relatively more complete genome (although at a higher cost). The genomes of 10 strains of *Pythium insidiosum* (the causative agent of the difficult-to-treat infectious disease, namely pythiosis, in humans and various animals worldwide [6, 7]) were sequenced using the Illumina short-read platform, resulting in up to sixty thousand contigs and thus incompletely assembled genome data [8–13]. This study aims to obtain a better-quality genome of *P. insidiosum* using the PacBio long-read. The *P. insidiosum* strain Pi-S was selected because it has been widely referred to in recent immunological, genomic, transcriptomic, and proteomic studies [8, 14–20]. The short-read version of the strain Pi-S genome data shows 53.2 Mb in size and comprises 1,192 contigs (average length: 44,664 bases; *N*_50_: 146,252 bases), 52% G-C content, 10% N composition, and 14,962 open reading frames (ORFs) [8]. When it becomes available, better-quality genome data of *P. insidiosum* can promote an in-depth genetic exploration and a better understanding of this filamentous microorganism’s biology, evolution, and pathogenicity. Such advances could lead to the development of a clinical application for preventing, diagnosing, and treating the disease caused by this devastating pathogen.

## Data description

Genomic DNA (gDNA) was obtained from 7-day-old liquid culture of *P. insidiosum* (strain Pi-S) using our previously described protocol [21]. Harvested hyphae (1,000 mg wet weight) were ground in a mortar after adding liquid nitrogen. Resulting hyphal power was moved to a sterile 50-mL tube containing 5 ml of the extraction solution [250 mM NaCl, 100 mM Tris–HCl (pH 8.0), 100 mM ethylenediaminetetraacetic acid (pH 8.0), and 1% sodium dodecyl sulfate] and RNase A (final concentration: 50 µg/ml). The cell lysate was incubated (with gentle inversion) at 37 °C for 2 h before adding proteinase K (final concentration: 50 µg/mL). After the sample was incubated at 56 °C overnight, the supernatant was collected by centrifugation (10,000 xg) at room temperature for 30 min, mixed with an equal volume of phenol:chloroform:isoamyl alcohol (25:24:1) solution, gently inverted for 15 min, and centrifuged (2,000 xg) at 25 °C (room temperature) for 30 min. The resulting aqueous phase was moved to a new 50-ml tube, mixed with the same volume of isopropanol, gently inverted 10 times, and centrifuged (2,000 xg) at 25 °C for 5 min. A resulting gDNA pellet was collected, washed with 70% ethanol, air dried, and resuspended in 5 mM Tris-HCl (pH 8.0). Extracted gDNA was kept at -20 °C and transported the following day to the National Omics Center, National Science and Technology Development Agency, Pathum Thani, Thailand, for genome sequencing.

Genome sequencing was done following the method of Shearman et al. [22]. In brief, a *P. insidiosum* gDNA sample was purified using the Ampure PB beads (Pacific Biosciences, Menlo Park, USA) and checked for DNA integrity using the Pippin Pulse Electrophoresis System (Sage Science, Beverly, USA). The gDNA sample (10 ng) was torn using a Covaris gTube (4,500 rpm for 2 min) for library preparation (using a ‘0.75%DF Marker S1 high-pass 15–20 kb’ BluePippin cassette with 12-50 kb selection) according to the Pacific Biosciences protocol (20 kb Template Preparation Using BluePippin Size-Selection). Genome sequencing was conducted on the PacBio RSII. Raw reads with a length of at least 20 kb were defined as seed reads, which were corrected by shorter reads (sizes < 20 kb) using the Pacific Biosciences SMRT analysis software v2.3.0 (the RS_PreAssembler.1 protocol with default settings).

PacBio-derived corrected reads (2,632.0 Mb) were assembled using CANU (v2.1) [23], a software that shows an ability to produce highly contiguous assemblies. Three rounds of polishing were carried out using ARROW [24] (SMRT link version 5.0.1). The first initial assemblies were aligned with the original raw PacBio reads using pbmm2 (v1.2.1) (https://github.com/PacificBiosciences/pbmm2). The resulting alignment was then used for calling the consensus sequence using ARROW. Gene prediction was made with the Funannotate pipeline (v1.7.4) (https://github.com/nextgenusfs/funannotate). As a result, a total of 840 contigs (maximum length: ~1.3 Mb; minimum length: 1,294 bases; *N*_50_: 229.9 Kb; *L*_50_: 70) were obtained. The sequence assembly showed a total genome length of 66.7 Mb (178x coverage) containing 57.2% G-C content and 20,375 ORFs. The genome completion estimated using BUSCO (v4.0.2) [25] and the Fungi Ogb10 dataset (containing 250 conserved eukaryotic genes) was 85.5%. All contig sequences have been deposited in the NCBI database under the accession numbers BBXB02000001 - BBXB02000840 [26] (Table 1).

**Table 1:** Overview of data files/data sets

Label	Name of data file/data set	File types (file extension)	Data repository and identifier (DOI or accession number)
Data file 1	*P. insidiosum* strain Pi-S, whole genome shotgun sequencing project	FASTA	BBXB02000001 - BBXB02000840 (https://identifiers.org/ncbi/insdc:BBXB00000000.2) [26]

## Limitations

We used the PacBio long-read NGS platform to improve the genome quality of *P. insidiosum* strain Pi-S. Compared with the short-read NGS platforms (such as Illumina), genome sequencing employing the long-read platforms (such as PacBio) provides a more complete genome sequence but is a relatively more expensive technique and shows a higher base error rate.

## Data Availability

The draft genome sequence of the *P. insidiosum* strain Pi-S comprising 840 contigs (accession numbers BBXB02000001 - BBXB02000840 [26]) is publicly available in the NCBI database.

## Abbreviations

DNA: Deoxyribonucleic acid.
gDNA: Genomic deoxyribonucleic acid.
NCBI: National Center for Biotechnology Information.
NGS: Next-generation sequencing.
OD: Optical density.
ORF: Open reading frame.
